# Rigid Neuroendoscopy Assisted Hematoma Resection Reduces the Recurrence Rate of Chronic Subdural Hematoma With Mixed Density: A Retrospective Analytic Cohort Study

**DOI:** 10.3389/fsurg.2022.789118

**Published:** 2022-02-25

**Authors:** Huangyi Fang, Zhongding Zhang, Yiru Liu, Lingfei Wang, Yue Yang, Shize Li, Xiepan Jing, Guanghui Bai, Hansong Sheng

**Affiliations:** ^1^The Second School of Medicine, Wenzhou Medical University, Wenzhou, China; ^2^Department of Neurosurgery, The Second Affiliated Hospital of Wenzhou Medical University, Wenzhou, China; ^3^Department of Neurosurgery, People's Hospital of Bayingolin Mongol Autonomous Prefecture, Korla, China; ^4^Department of Radiology, The Second Affiliated Hospital of Wenzhou Medical University, Wenzhou, China

**Keywords:** chronic subdural hematoma, CT density value, burr-hole craniotomy, neuroendoscopy, outcome, recurrence

## Abstract

**Background:**

The mixed density hematoma (MDH) has a high recurrence rate in chronic subdural hematoma (CSDH). This study adopted rigid neuroendoscopy assisted hematoma resection to evacuate CSDH and investigated its efficacy as compared with the traditional burr-hole craniostomy (BHC) in CSDH with mixed density.

**Methods:**

A retrospective cohort study was conducted at two centers between January 2015 and December 2020. The data of 124 patients who underwent BHC for CSDH with mixed density were collected and analyzed. A total of 41 patients underwent rigid neuroendoscopy assisted hematoma resection (neuroendoscopy group) and 83 patients were treated by the traditional BHC (control group). Follow-ups were conducted 6 months after the surgery.

**Results:**

There was no significant difference in the baseline characteristics and preoperative CT features between the two groups (*p* > 0.05). The neuroendoscopy group had a lower recurrence rate than the control group (*p* = 0.043). Besides the neuroendoscopy group had a higher rate of hematoma evacuation (*p* < 0.001), less pneumocephalus volume (*p* < 0.001), shorter hospital stay (*p* < 0.001) and better Markwalder score (*p* < 0.001) than the control group within 24–48 h after operation. However, there was no significant difference between the two groups in the incidence of pneumocephalus, Markwalder score (at discharge and 6 months after surgery) and mortality. Moreover, the operation time was longer in the neuroendoscopy group (*p* < 0.001).

**Conclusions:**

When compared with the traditional BHC, rigid neuroendoscopy assisted hematoma resection can better reduce the recurrence rate of CSDH with mixed density. Also, it surpassed the results obtained from BHC in reducing the volume of pneumocephalus, improving hematoma evacuation rate, promoting short-term neurological recovery, and shortening hospital stays.

## Introduction

Chronic subdural hematoma (CSDH) is one of the most common neurosurgical diseases that accounts for about 10% of all intracranial hematomas. Its incidence has been reported to range from 1 to 48 per 100,000 persons per year in different areas (1, 2). With the development of the aging society, the incidence of CSDH is also arising (3). A new meta-analysis indicated that the recurrence rate of CSDH is 14.4% (4). Among the many factors affecting CSDH recurrence, the density of the hematoma was reported as an independent predictor for recurrence. The patients with mixed density hematoma which means repeated bleeding have a higher recurrence rate (5).

In general, asymptomatic patients are managed with conservative measures. But for patients with symptoms of brain compression, surgical treatment is the most effective and widely-used therapy for CSDH, including twist-drill craniostomy, BHC, and craniotomy (6). Although most cases of CSDH resolve after BHC, its recurrence remains a problem (7). With the development of endoscopic neurosurgical techniques, the application of rigid neuroendoscopy in the treatment of CSDH has shown unique advantages and satisfactory efficacy over recent years (8). This retrospective study was carried out to compare the rigid neuroendoscopy assisted hematoma resection with the traditional BHC to investigate their curative effects on the CSDH with mixed density.

## Materials and Methods

### Patients

**Figure 1:**
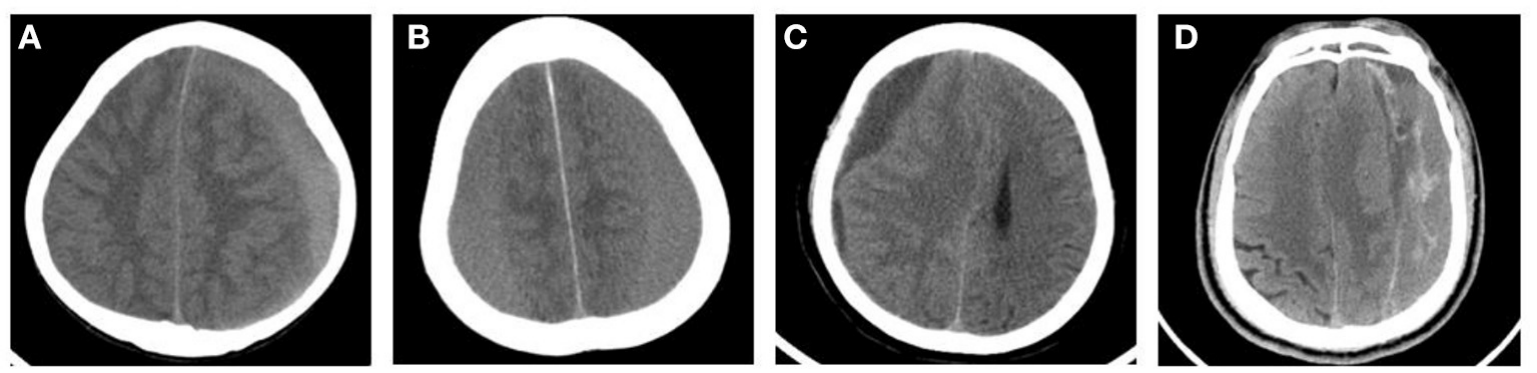
Typical CT images of the classification of CSDH according to the hematoma density. **(A)** High-density hematoma (HDH). **(B)** Iso-density hematoma (IDH). **(C)** Low-density hematoma (LDH). **(D)** Mixed-density hematoma (MDH).

We retrospectively analyzed the medical records of all patients undergoing CSDH surgical treatment at the Department of Neurosurgery of two hospitals (the Second Affiliated Hospital of Wenzhou Medical University and People's Hospital of Bayingolin Mongol Autonomous Prefecture) between January 2015 and December 2020. This study was approved by the Ethics Committee of the two hospitals. We classified the density of the subdural hematomas into four groups based on CT findings as previously reported: low-density hematoma (LDH) [ <25 Hounsfield units (HU)], iso-density hematoma (IDH) (25–35 HU), high-density hematoma (HDH) (>35 HU), and mixed-density hematoma (MDH) (9) (Figure 1).

Inclusion criteria were as follows: (1) CT showed initial CSDH with mixed density; (2) BHC or rigid neuroendoscopy assisted hematoma resection treatment. Exclusion criteria were as follows: (1) history of antiplatelet or anticoagulant medication; (2) CSDH recurrence; (3) history of craniocerebral surgery. A total of 141 patients met the inclusion criteria, but nine patients had a history of antiplatelet or anticoagulant medication, six patients had a history of craniocerebral surgery, and two patients with recurrent CSDH. Finally, we collected the medical records of 124 patients who met the criteria.

Then the patients were divided into the control group and neuroendoscopy group, 83 cases of CSDH were treated by BHC in the control group and 41 cases were treated by BHC with rigid neuroendoscopy in the neuroendoscopy group. The choice of surgical technique for the patient was determined by the responsible consultant.

### Clinical Data

We collected clinical data consisting of the patients' baseline characteristics and CT features. Baseline characteristics included the patients' age, gender, smoking status, alcoholic consumption status, present, complaints, trauma history, medical history, drug history, and Glasgow Coma Scale on admission. The CT images were analyzed with the 3D slicer (version 4.10.2, https://www.slicer.org/). In addition, we roughly judged the site of the hematoma by the corresponding position on the opposite side of the hematoma. The patient's hematoma often contained multiple sites, so these sites were counted separately in Table 1.

**Table 1:** Baseline characteristics.

**Items**	**Neuroendoscopy group (*n* = 41)**	**Control group (*n* = 83)**	***P*-value**
Age(year)	64.3 (59.67–68.87)	65.5 (61.26–61.28)	0.700
Male	34 (82.9%)	66 (79.5%)	0.651
Smoking	13 (31.7%)	25 (30.1%)	0.234
Alcoholic	8 (19.6%)	18 (21.7%)	0.641
Symptoms
Symptomless	1 (2.4%)	1 (1.2%)	1.000
Headache	20 (47.9%)	45 (54.2%)	0.323
Dizziness	13 (31.8%)	21 (25.3%)	0.084
Nausea/vomiting	7 (17.1%)	16 (19.3%)	0.696
Limb weakness	14 (34.1%)	30 (36.1%)	0.397
Cognitive decline	7 (17.1%)	9 (10.8%)	0.108
Speech impairment	1 (2.4%)	4 (4.8%)	1.000
Trauma history	23 (65.9%)	46 (55.4%)	0.068
Underlying disease
Hypertension	21 (51.2%)	40 (48.2%)	0.063
Diabetes	4 (12.9%)	12 (14.5%)	1.000
Coronary heart disease	5 (12.2%)	7 (8.4%)	0.234
Pulmonary tuberculosis	0 (0%)	2 (2.4%)	1.000
Atrial fibrillation	3 (7.3%)	5 (6.0%)	0.789
Malignant neoplasm history	5 (12.2%)	7 (8.4%)	0.234
Neurological score on Admission
Glasgow Coma Scale			1.000
13–15	38	78	
9–12	3	5	
3–8	0	0	

### Calculation of the Volume of Hematoma and Pneumocephalus

Firstly, we used the segmentations module to create a segment in the axial CT image of the head, choosing the hematoma/pneumocephalus area with the threshold tool. Secondly, we modified the edge of the hematoma/pneumocephalus and filled the bubble inside to confirm the precision of the segment. Lastly, the 3D model was reconstructed based on this segment, which showed the volume of hematoma/pneumocephalus.

### Measurement of Mid Line Shift

**Figure 2:**
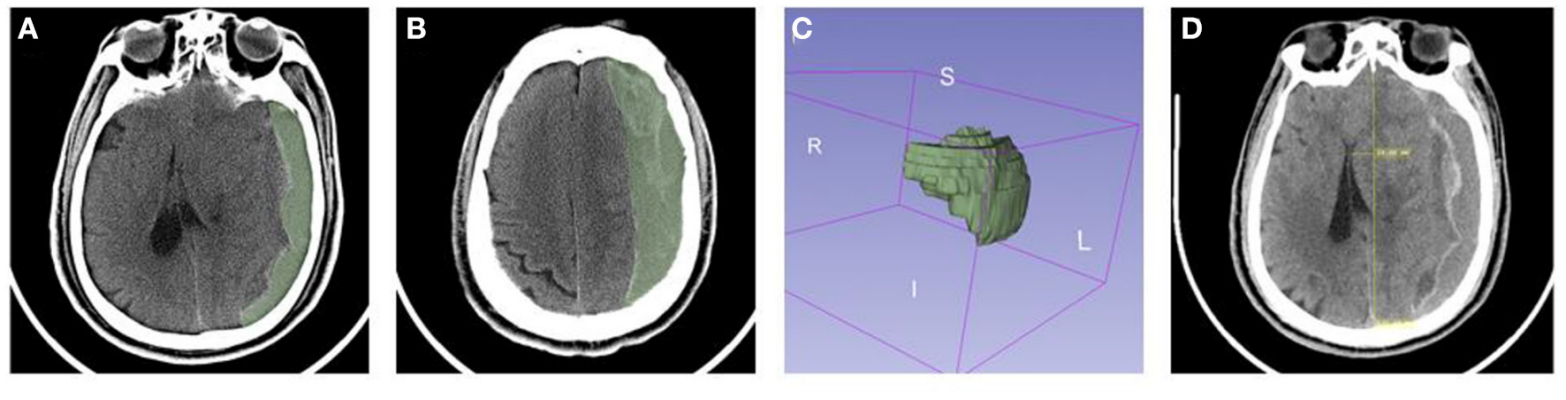
The CT images of CSDH with mixed density were analyzed with 3D slicer. **(A–C)** Calculation of the volume of hematoma. **(D)** Measurement of mid line shift.

At first, we used the reformat module to adjust the axial CT image into the standard anatomical position, then used the ruler tool to draw a line connecting the frontal median ridge and the parietal bone, and projected the line from 3D to 2D. Next we observed each layer of CT image to figure out the maximum site location. Meanwhile, we drew a vertical line to the base line, whose length could be regarded as midline shift (Figure 2).

### Surgical Procedures

All patients underwent surgery under general anesthesia.

The control group used the traditional BHC procedure. According to preoperative CT positioning, we chose the location of the incision at the thickest layer of the hematoma. A single 2 cm burr hole was drilled with a highspeed drill. The dura mater was coagulated with bipolar coagulation forceps and opened with a cruciate incision. After partial drainage, the hematoma cavity was rinsed repeatedly with normal saline until the retracted rinse fluid was clear. Then an 8 mm silastic catheter was inserted into the cavity for drainage. The drainage bag was connected with the catheter and fixed at the same height as the patient's tragus for continuous drainage.

**Figure 3:**
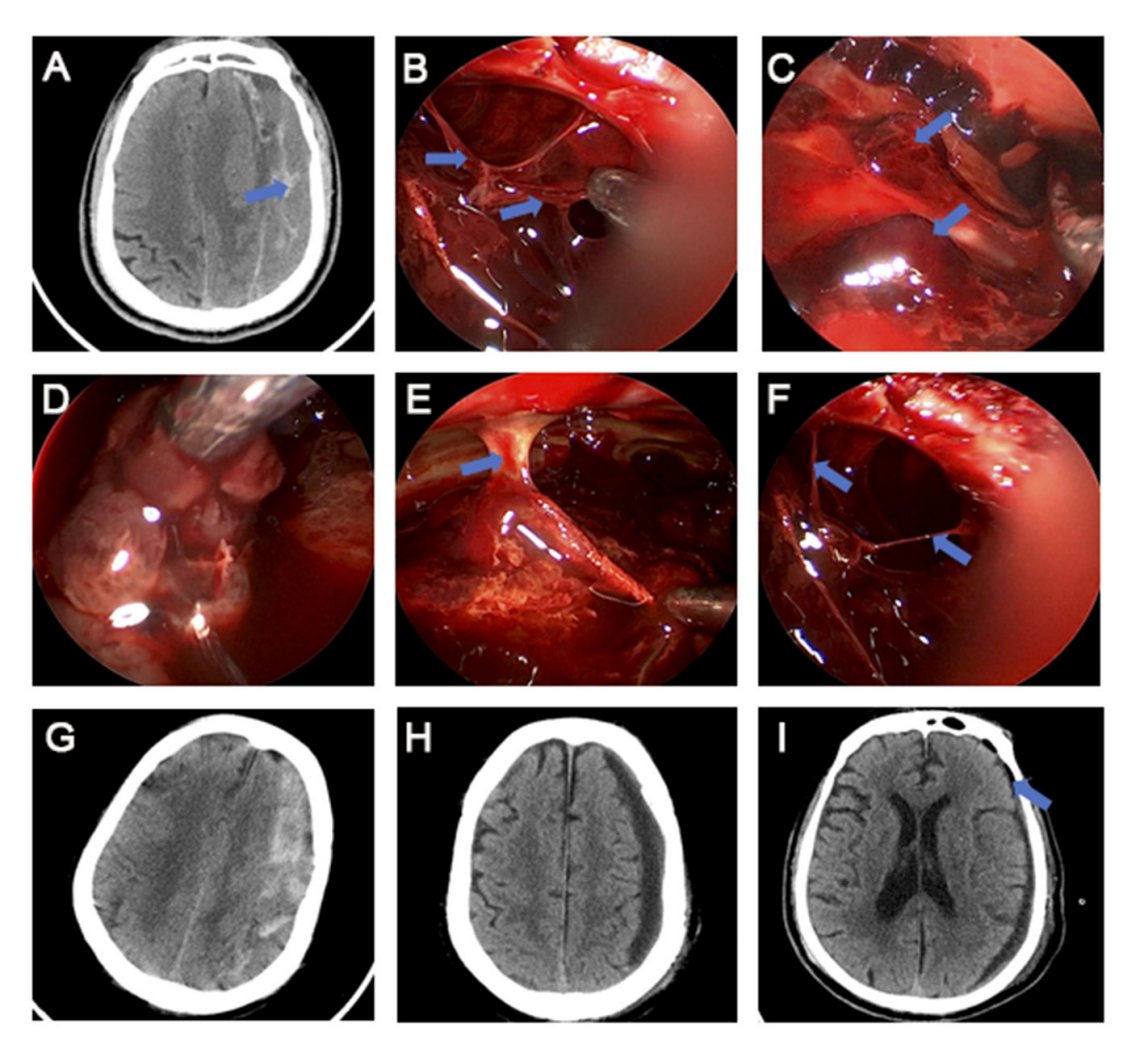
Preoperative and postoperative CT images and intraoperative images of rigid neuroendoscopy of CSDH. **(A,B)** Multiple-septum (blue arrow) of the hematoma. **(C)** Blood clots (blue arrow) that adhere closely to the capsule. **(D)** Suction of the incomplete liquefied hematoma. **(E)** A columnar trabecular structure (blue arrow) formed by the adhesion of the parietal and visceral envelope. **(F)** Bridging veins (blue arrow) in the hematoma cavity. **(G,H)** Comparison of preoperative **(G)** and postoperative **(H)** CT in a patient with the mixed density CSDH treated with rigid neuroendoscopy. **(I)** Little postoperative pneumocephalus (blue arrow) after rigid neuroendoscopy treatment.

The neuroendoscopy group underwent a different surgical procedure from the traditional BHC procedure. The hematoma cavity was observed with rigid neuroendoscopy after irrigation with normal saline. We used an aspirator to remove the hematoma. For the hematomas with multiple compartments, the hematomas in each compartment were successively removed after the stoma was performed. The blood clots that adhered closely to the capsule was removed from the capsule and sucked out. Active bleeding on the inner surface of the capsule can be stopped by bipolar electrocoagulation and gauze compression in turn. The bridging veins with high tension observed under rigid neuroendoscopy were cut off after electrocoagulation. Finally, the hematoma cavity was rinsed with saline, and the 8 mm silicone tube was indwelled to the lowest part of the hematoma cavity (Figure 3).

### Postoperative Management and Outcome Measures

The CT scan of patient's head was performed within 24–48 h after operation. The drainage tube was removed after the hematoma secretions have dried up and confirmed adequate treatment by CT.

The primary outcome measure was recurrence which we defined as a symptomatic recurrence that resulted in a previous ipsilateral hematoma requiring re-operative removal (10).

The secondary outcome measures included operation time, postoperative hematoma volume and hematoma evacuation rate within 24–48 h after operation, the occurrence of pneumocephalus and pneumocephalus volume, length of postoperative hospitalization, neurological function and mortality after surgery. We defined pneumocephalus as the postoperative volume of intracranial gas >3 ml on either side. The postoperative neurological function was evaluated with the Markwalder Score. The score of 0–1 indicated a good prognosis.


$$\begin{array}{l} \text{Hematoma evacuation rate}=\;\frac{\text{Hematoma volume }-\text{ Postoperative hematoma volume}}{\text{Hematoma volume}} \end{array}$$

### Statistical Analysis

All statistical analyses were conducted using SPSS software version 21 (IBM Corp., Armonk, New York, USA). Quantitative data was described by mean (±standard dewell tion), and differences between variables were compared using the Student *t*-test. Qualitative data was described by number (%), and differences between variables were evaluated using the *X*^2^ test. *p* < 0.05 was considered statistically significant.

## Results

In our study, there was no significant difference between the two groups in terms of the baseline characteristics (Table 1) and preoperative CT features (Table 2) (*p* > 0.05). In the baseline characteristics of the two groups, headache was the most common symptom and hypertension was the most common underlying disease. In the preoperative CT features of the two groups, the most common side of hematoma was the left, and the most common site was the temporal.

**Table 2:** Preoperative CT features.

**Items**	**Neuroendoscopy group (*n* = 41)**	**Control group (*n* = 83)**	***P*-value**
Hematoma volume (ml)	119.76 (109.31–130.03)	117.47 (106.30–128.64)	0.773
Hematoma thickness(mm)	20.32 (18.56–22.07)	22.35 (20.61–24.10)	0.102
Side			0.813
Left	17 (41.5%)	30 (44.6%)	
Right	14 (34.1%)	29 (34.9%)	
Bilateral	10 (24.4%)	24 (28.9%)	
Site
Frontal	15 (36.6%)	38 (45.8%)	0.343
Parietal	22 (53.7%)	51 (61.4%)	0.442
Temporal	30 (73.2%)	67 (80.7%)	0.361
Occipital	2 (4.9%)	6 (7.2%)	1.000
The midline shift in	8.80 (7.84–9.77)	7.80 (6.93–8.66)	0.122
unilateral hematomas (mm)			

Under the rigid neuroendoscopy, the CSDH with mixed density in CT was heterogeneous in texture, including liquid stale blood, incomplete liquefied blood clot and multiple-septum. For high tension bridging veins, we performed electrocoagulation and resection under direct vision (Figure 3).

The primary and secondary outcome measures were listed in Table 3. The mean operation time in the neuroendoscopy group (95.20 min) was longer than in the control group (71.36 min) (*p* < 0.001). The mean hematoma evacuation rate in the neuroendoscopy group (96%) was higher than in the control group (89%) (*p* = 0.03). The mean pneumocephalus volume in the neuroendoscopy group (5.58 ml) was less than in the control group (13.67 ml) (*p* < 0.001). However, there was no significant difference in the incidence of pneumocephalus between the two groups (*p* = 0.567). The mean duration of postoperative hospital stay in the neuroendoscopy group (8.54 day) was lower than in the control group (11.84 day) (*p* = 0.03). Follow-up results showed that the recurrence rate in the neuroendoscopy group (1/41, 2.4%) was lower than that in the control group (14/83, 16.9%) within 6 months after surgery. The ratio of Markwalder Score of 0–1 in the neuroendoscopy group (30/41, 73.2%) was lower than in the control group (43/83, 51.8%) on the first day after surgery (*p* = 0.03). But there was no significant difference between the two groups in the ratio of Markwalder Score of 0–1 at discharge (*p* = 0.938) and 6 months after surgery (*p* = 1.000). Moreover, there was no difference in mortality between the two groups within 6 months after surgery (*p* = 1.000).

**Table 3:** Postoperative outcomes.

**Items**	**Neuroendoscopy group (*n* = 41)**	**Control group (*n* = 83)**	***P*-value**
Operation time (min)	95.20 (87.39–103.01)	71.36 (62.41–80.31)	**<0.001**
Postoperative CT features (24–48h)			
Postoperative hematoma volume (ml)	4.71 (3.63–5.80)	12.77 (10.35–15.18)	**<0.001**
Volume of hematoma evacuation (ml)	114.96 (104.81–125.11)	104.70 (94.63–114.77)	0.153
Hematoma evacuation rate (%)	96 (95–97)	89 (87–91)	**<0.001**
Pneumocephalus	21 (51%)	37 (46%)	0.567
Pneumocephalus volume (ml)	5.58 (4.31–6.85)	13.67 (4.25–23.09	**<0.001**
Duration of postoperative hospital stay (day)	8.54 (7.74–9.34)	11.84 (10.45–13.24)	**<0.001**
Markwalder Score (0–1)			
On postoperative day	30 (73.2%)	43 (51.8%)	**0.023**
At discharge	37 (90.2%)	73 (88.0%)	0.938
At 6 months	39 (95.1%)	78 (94.0%)	1.000
Recurrence rate (6 months)	1 (2.4%)	14 (16.9%)	**0.043**
Mortality (6 months)^*^	0	1 (1.2%)	1.000

* *The patient in the control group died of post-traumatic cerebral hemorrhage*.

## Discussion

Previous studies have reported the mixed density as an independent risk factor for recurrence of CSDH (11, 12). With the development of endoscopic technology, the advantages of its visualization help the surgeon to directly observe and clean up the hematoma, which may be conducive to improving the prognosis of patients. Our study compared the efficacy of rigid neuroendoscopy assisted hematoma resection and BHC in the treatment of CSDH with mixed density and showed that the former can reduce the recurrence rate and improve prognosis of patients. Such results were in line with our assumptions and reflected the advantages of neuroendoscopy.

With the aging of population, the incidence of CSDH is increasing. Unlike acute subdural hematoma, the CSDH, which is prevalent in the elderly, develops 3 weeks or more after head injury (13, 14). Trauma is the main cause of CSDH, leading to the rupture of the bridging vein or cerebral cortex blood vessel, followed by accumulation of blood in the subdural space (6). Inflammatory response, angiogenesis, subdural effusion evolution and local coagulation disorders contribute to CSDH as well (15, 16).

Among many factors associated with the recurrence of CSDH, MDH is an independent risk factor for the recurrence and reoperation of CSDH (12). The possible reason for this is that the hematoma is not completely liquefied in MDH, which makes the drainage tube easy to block after surgery and successively give rise to the poor drainage. Next, the residual hematoma would cause a widening of space between the dura mater and brain tissue, which may tear bridging vein and cause re-bleeding (5, 11).

For patients with symptoms or radiographic findings of cerebral compression, surgical treatment is a better option (17). A survey showed that BHC is the most commonly used among various surgical treatments for CSDH (18). However, its recurrence remains a problem, especially in patients kept upright. Irrigation is an important part of surgery, previous study got a better outcome by modifying irrigation methods, which may be related to the flushing away of more coagulation factors and inflammatory factors (19). Incomplete liquefaction of hematoma easily results in poor drainage after surgery. With the development of endoscopic technology, the hematoma can be seen more directly. We cleared the septal cavity and cut off bridging veins with high tension under rigid neuroendoscopy, which promoted hematoma expulsion and prevented rebleeding. 3D modeling was adopted to calculate hematoma volume in 3D Slicer software (Figure 2A), which had been proved to be more accurate than the traditional Tada formula method in hematoma volume calculation (20, 21). In our study, rigid neuroendoscopy assisted hematoma resection indicated a higher hematoma clearance compared with traditional BHC. However, the operation time has also increased at the same time.

Previous findings revealed that pneumocephalus increases the recurrence rate of CSDH and leads to deterioration of the neurological function (22). This may have been due to the bridging veins in the hematoma cavity being repeatedly lacerated by floating air bubbles as the patient's head shook (23). All patients had at least some intracranial gas after surgery, so we defined the pneumocephalus as the postoperative volume of intracranial gas >3 ml on either side. This study found that rigid neuroendoscopy assisted hematoma resection can reduce the volume of the pneumocephalus compared to traditional BHC. This may be due to disruption of the Multiple-septum of the hematoma, which helps to maintain the full volume of hematoma cavity and prevent gas entry. However, rigid neuroendoscopy assisted hematoma resection didn't significantly reduce the incidence of pneumocephalus.

The neurological function score of the neuroendoscopy group on the first day after surgery was obviously better than that of the control group. It indicated that the use of rigid neuroendoscopy was helpful to improve short-term neurological function recovery, which led to shorter postoperative hospital stays. We presume that this result is attributed to a more efficient hematoma evacuation. However, there was no difference in neurological scores between the two groups at discharge and 6 months after surgery, which may suggest that the use of the rigid neuroendoscopy had little effect on long-term neurological function recovery.

Compared with the control group, the neuroendoscopy group had a lower recurrence rate in our study. Hematoma evacuation rate and the occurrence of pneumocephalus are independent factors for the recurrence of CSDH (24). The neuroendoscopy group performed better in these aspects.

There are some limitations in this study. First, this research is a retrospective study; therefore, the possibility of selection bias in surgical technique cannot be denied. Second, this research is limited by different centers, although we have no clear indications that this will affect results regarding recurrence or complications.

Taken together, rigid neuroendoscopy-assisted hematoma evacuation was more advantageous than traditional BHC in the treatment of mixed density CSDH in our study. However, in order to avoid the risk of bias from the retrospective study, prospective studies based on large populations is needed to further confirm these results in the future.

## Conclusion

When compared with the traditional BHC, rigid neuroendoscopy assisted hematoma resection can better reduce the recurrence rate of CSDH with mixed density and the volume of pneumocephalus, improve the clearance rate of hematoma, promote the recovery of short-term neurological function and shorten the length of hospital stay.

These findings suggest that rigid neuroendoscopy may be a better therapeutic option in BHC for mixed density CSDH.

## Data Availability Statement

The original contributions presented in the study are included in the article/supplementary material, further inquiries can be directed to the corresponding author/s.

## Ethics Statement

The studies involving human participants were reviewed and approved by Medical Ethics Committee, Yuying Children's Hospital, the Second Affiliated Hospital of Wenzhou Medical University. Written informed consent for participation was not required for this study in accordance with the national legislation and the institutional requirements.

## Author Contributions

HF and HS: conceived the study. HF, YL, ZZ, and LW: designed the study. HF, YL, YY, and SL: finished the data collection. HF, ZZ, YY, and XJ: analyzed and interpreted the data and drafted the article. GB and HS: checked the analyses and the interpretations. HF, ZZ, YL, LW, and HS: reviewed the manuscript critically. All authors approved the manuscript for publication.

## Conflict of Interest

The authors declare that the research was conducted in the absence of any commercial or financial relationships that could be construed as a potential conflict of interest.

## Publisher's Note

All claims expressed in this article are solely those of the authors and do not necessarily represent those of their affiliated organizations, or those of the publisher, the editors and the reviewers. Any product that may be evaluated in this article, or claim that may be made by its manufacturer, is not guaranteed or endorsed by the publisher.
